# Mixotrophic growth of bacteriochlorophyll *a*-containing members of the OM60/NOR5 clade of marine gammaproteobacteria is carbon-starvation independent and correlates with the type of carbon source and oxygen availability

**DOI:** 10.1186/1471-2180-13-117

**Published:** 2013-05-24

**Authors:** Stefan Spring, Thomas Riedel

**Affiliations:** 1 Leibniz Institute DSMZ – German Collection of Microorganisms and Cell Cultures, Inhoffenstr. 7B, Braunschweig 38124, Germany; 2 Helmholtz-Centre for Infection Research (HZI), Research Group Microbial Communication, Inhoffenstr. 7, Braunschweig 38124, Germany; 3 Present address: Observatoire Océanologique de Banyuls, Université P. et M. Curie, UMR-CNRS 7621, Laboratoire Arago, 66650 Banyuls-sur-Mer, France

**Keywords:** Photoheterotrophy, Photophosphorylation, *Roseobacter* clade, Carbon starvation, Coastal marine environment

## Abstract

**Background:**

Populations of aerobic anoxygenic photoheterotrophic bacteria in marine environments are dominated by members of the *Roseobacter* lineage within the *Alphaproteobacteria* and the OM60/NOR5 clade of gammaproteobacteria. A wealth of information exists about the regulation of pigment production and mixotrophic growth in various members of the *Roseobacter* clade, but a detailed knowledge about aerobic bacteriochlorophyll *a*-containing gammaproteobacteria is still limited to one strain of the species *Congregibacter litoralis*.

**Results:**

The production of photosynthetic pigments and light-dependent mixotrophic growth was analysed in *Luminiphilus syltensis* DSM 22749^T^, *Chromatocurvus halotolerans* DSM 23344^T^ and *Pseudohaliea rubra* DSM 19751^T^, representing three taxonomically diverse strains of bacteriochlorophyll *a*-containing gammaproteobacteria affiliated to the OM60/NOR5 clade. In these strains the expression of a photosynthetic apparatus depended mainly on the type of carbon source and availability of oxygen. The effect of illumination on pigment expression varied significantly between strains. In contrast to *Chromatocurvus halotolerans,* pigment production in *Luminiphilus syltensis* and *Pseudohaliea rubra* was repressed by light of moderate intensities, probably indicating a higher sensitivity to light-induced oxidative stress. The efficiency of using light for mixotrophic growth did not correlate with the cellular level of photosynthetic pigments, but depended mainly on the type of metabolized substrate with malate being the optimal carbon source in most cases.

**Conclusions:**

Oligotrophic growth conditions or carbon limitation were not required for light-dependent mixotrophic growth in members of the OM60/NOR5 clade. The ability of using light as energy source and the fine tuning of photosynthesis gene expression depended mainly on the type of carbon source and oxygen availability, which indicates that the regulation of pigment production is controlled by the cellular redox state. While light has the main impact on the regulation of photosynthetic pigments in photoheterotrophic representatives of the *Roseobacter* lineage this was not the case in strains of the OM60/NOR5 clade.

## Background

Aerobic anoxygenic photoheterotrophic bacteria are found in large numbers in upper ocean waters and marine sediments [1-3]. Populations of this functional group in marine ecosystems are dominated by representatives belonging to the *Roseobacter* clade within the class *Alphaproteobacteria* and the OM60/NOR5 clade within the *Gammaproteobacteria*[4,5]. Due to their high abundance in oceans, aerobic anoxygenic photoheterotrophs can play a significant role in the marine carbon cycle. It was estimated that up to 5.7% of the total phototrophic energy flow in open ocean waters could rely on bacteriochlorophyll *a* (BChl *a*)-based photophosphorylation [6,7]. The prevalence of aerobic anoxygenic photoheterotrophy in marine ecosystems is probably based on two reasons: First, the utilization of light for mixotrophic growth enhances biomass formation under conditions of carbon limitation and gives aerobic anoxygenic photoheterotrophs a selective advantage against obligate chemoheterotrophic bacteria. Secondly, utilization of solar energy by aerobic anoxygenic photoheterotrophs is largely independent from photoinhibition, which is caused by high light-intensities in surface waters and reduces the chlorophyll *a*-based photosynthetic activity of oxygenic photoautotrophs [6].

In order to verify both assumptions, it is of interest to elucidate which factors control the expression of the photosynthetic apparatus in cells of aerobic anoxygenic photoheterotrophs and how the energy yield generated by light-harvesting correlates with the environmental conditions. The regulation of pigment production and light-dependent growth in members of the *Alphaproteobacteria* has been analysed previously in numerous studies [8-13]. In most of these studies exposure to light was identified as major factor that negatively controls the expression level of photosynthetic pigments. In a study about the transcriptional response of *Dinoroseobacter shibae* to changing light regimes the repression of pigment synthesis in the light coincided with a response to oxidative stress that was traced back to the formation of singlet oxygen at the photosynthetic apparatus. It was assumed that in response to the oxidative stress caused by the interaction of light with photosynthetic pigments a repression of the photosynthetic pigment production is induced by the transcriptional modulator TspO [14]. In contrast, the corresponding knowledge about BChl *a*-containing aerobic gammaproteobacteria belonging to the OM60/NOR5 clade is still quite sparse due to the low number of available pure cultures and their fastidious growth in defined media. Previously, it was shown that in the aerobic gammaproteobacterium *Congregibacter litoralis* (*C. litoralis*) anoxygenic photophosphorylation depends on the carbon source and incubation conditions [15], but not on the carbon concentration, which is in contradiction to the finding of Cho et al. [16], who analysed the mixotrophic growth of the marine gammaproteobacterium HTCC2080 and found a positive correlation with very low nutrient concentrations. In another study a correlation of the pigment production in *Chromatocurvus halotolerans* (*C. halotolerans*) with the salinity of the used medium was found [17]. The reported results are however difficult to compare, because the experimental setups were not consistent. In order to broaden our knowledge on the mixotrophic growth behaviour of aerobic BChl *a*-containing gammaproteobacteria it would be therefore desirable to analyse various strains of this clade using the same study design. In the present work, three taxonomically diverse strains of the gammaproteobacterial OM60/NOR5 clade were analysed applying the same methods as developed previously for *C. litoralis*, so that the obtained results can be compared with existing data. The phylogenetic positions of these strains are as follows: *Luminiphilus syltensis* (*L. syltensis*) DSM 22749^T^ is affiliated to the NOR5-1 lineage of the OM60/NOR5 clade and related to the strain HTCC2080, *Pseudohaliea rubra* (*P. rubra*) DSM 19751^T^ is closely related to *C. litoralis* and belongs to the NOR5-3 lineage, whereas *C. halotolerans* DSM 23344^T^ is associated with the NOR5-3 branch, but does not belong to it [5]. The physiological and genotypic differences between these strains have been described in an accompanying paper by Spring et al. [18].

## Results and discussion

### The production of photosynthetic pigments is influenced by the type of carbon source and oxygen availability

**Figure 1:**
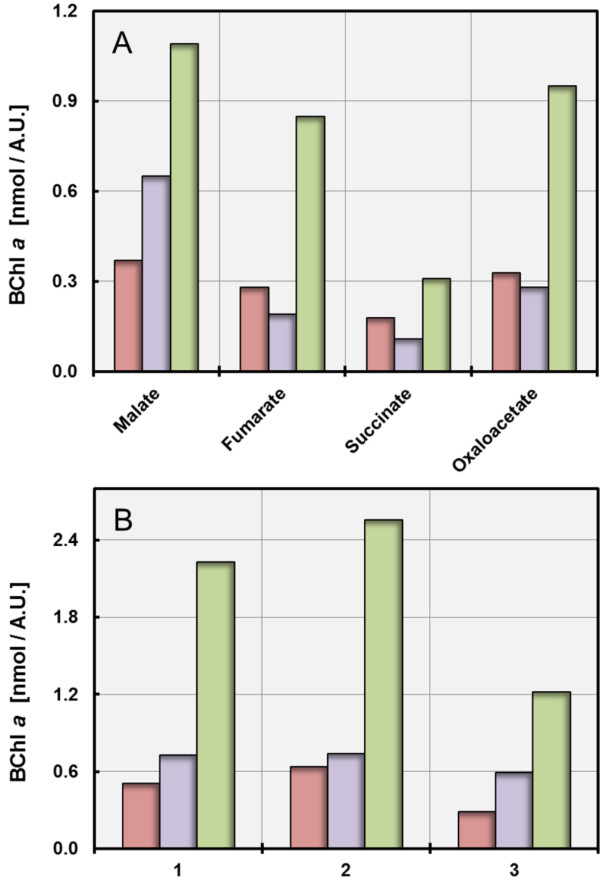
**Correlation of the production of photosynthetic pigments with the type and amount of carbon source in batch cultures.** Cultures were incubated under dim light with 12% (v/v) O_2_ in the headspace gas atmosphere. The amount of produced BChl *a* is symbolized by red bars for *L. syltensis* DSM 22749^T^, blue bars for *C. halotolerans* DSM 23344^T^ and green bars for *P. rubra* DSM 19751^T^. **A**. The effect of substrate reduction on pigment production is demonstrated by cultivation in defined media containing 10 mM of the respective carbon source. **B**. The dependence of pigment production on substrate concentration is shown by cultivation of *L. syltensis* DSM 22749^T^ in defined medium with 12% (v/v) O_2_ in the headspace gas atmosphere containing 2.5 mM pyruvate (1), 5.0 mM pyruvate (2) and 10.0 mM pyruvate (3) as carbon source. *C. halotolerans* DSM 23344^T^ and *P. rubra* DSM 19751^T^ were grown in defined medium containing 2.5 mM DL-malate (1), 5.0 mM DL-malate (2) and 10.0 mM DL-malate (3) as carbon source.

The amount of produced photosynthetic pigments in the type strains of *L. syltensis*, *C. halotolerans* and *P. rubra* was determined upon growth on different substrates in defined medium. In Figure 1A results obtained with intermediates of the citric acid cycle as carbon sources are shown. The highest production of photosynthetic pigments was achieved in all three strains with malate, whereas succinate yielded the lowest amount of pigments. This effect was most pronounced in *C. halotolerans* and less significant in *L. syltensis*. A similar correlation between carbon source and pigmentation was obtained in a previous study with *C. litoralis*[15], which indicates the consistency of this effect among various strains belonging to the OM60/NOR5 clade. Succinate is a more reduced substrate compared to malate or oxaloacetate, because the complete oxidation of succinate to CO_2_ results in a higher yield of reducing equivalents. Hence, it can be deduced that use of a highly reducing substrate inhibits the expression of photosynthetic pigments in photoheterotrophic strains of the OM60/NOR5 clade by the accumulation of reductants (*e.g.*, NADH), which affects the intracellular redox state. An influence of the reduction level of the substrate on the cellular redox poise of the facultatively anaerobic phototrophic bacterium *Rhodospirillum rubrum* was demonstrated by Grammel and Gosh [19], who concluded that in this species the substrate-dependent reduction of the ubiquinone pool has a main influence on the regulation of pigment production. A principal effect of substrate utilization on photoheterotrophic growth in the absence of a redox-balancing system could be also recently demonstrated by Laguna et al. [20]. They used ribulose-1,5-bisphosphate carboxylase/oxygenase (RuBisCO)-deletion strains of facultative anaerobic photoheterotrophic alphaproteobacteria as model organisms and could show that excess reductant produced by the assimilation of DL-malate led to a prevention of photoheterotrophic growth in mutant strains that were not able to consume reductant by CO_2_ fixation.

Numerous independent experiments were performed to determine the influence of oxygen availability and carbon concentration on pigment expression using media containing various amounts of carbon source and/or different concentrations of oxygen in the head space gas atmosphere. Similar results were obtained upon cultivation in closed serum bottles, if either the oxygen concentration was reduced at a constant substrate concentration or the substrate concentration increased at a constant oxygen concentration. Thus, the pigmentation in these strains depended on the carbon/oxygen balance. For each analysed strain results of a representative experiment are shown in Figure 1B. It can be deduced that in all tested strains pigment expression is repressed when oxygen is limiting growth. The same result was obtained previously with *C. litoralis*[15]. Hence, the reduction of pigment expression in the presence of growth-limiting oxygen concentrations is a conserved trait in all BChl *a*-containing members of the OM60/NOR5 clade studied so far. On the other hand, there was some variability in the effect of an oxygen excess or carbon limitation on pigmentation among different strains upon growth in batch cultures. A high oxygen to carbon ratio decreased the production of pigments in *C. litoralis*[15], *P. rubra* and *L. syltensis,* whereas it had no significant negative effect on the pigmentation of *C. halotolerans*. Nevertheless, a stimulation of pigment production in the tested strains was never observed by a lowering of the concentrations of carbon sources to 1 – 2 mM in order to imitate oligotrophic growth conditions. In addition, amounts of the essential nutrients ammonium, phosphate and iron were always in excess, which did not seem to have a negative effect on pigment production, at least in batch cultures.

Interestingly, no effect of substrate utilization or oxygen concentration on pigment production was found in several members of the *Roseobacter* clade that were studied in this respect [10,11], which may be due to the use of different regulatory pathways or a more stable cellular redox state in these bacteria compared to members of the OM60/NOR5 clade.

### Utilization of light for mixotrophic growth depends on the metabolized substrate

**Figure 2:**
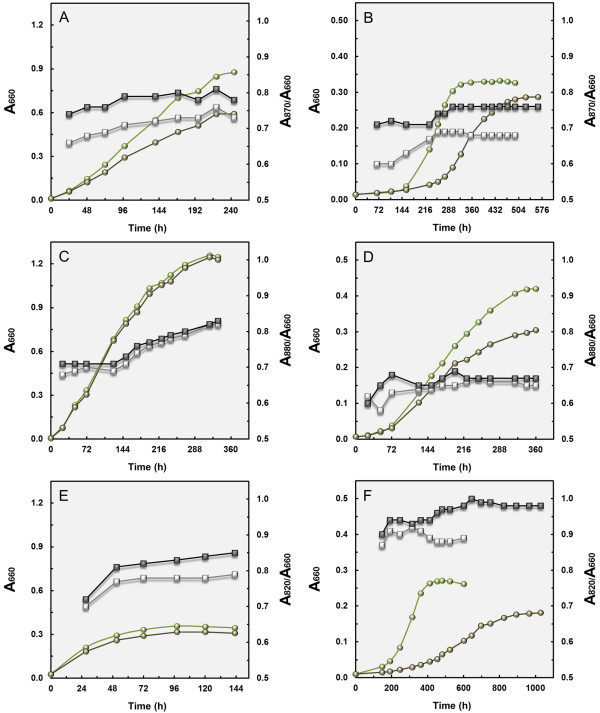
**Growth curves in light and darkness.** Growth curves were determined in duplicate and symbols represent means of both measurements. Circles represent A_660nm_ values. Squares symbolize A_870nm_/A_660nm_ values in strain *L. syltensis* DSM 22749^T^, A_880nm_/A_660nm_ values in *C. halotolerans* DSM 23344^T^ and A_820nm_/A_660nm_ values in *P. rubra* DSM 19751^T^. Light green circles and open squares indicate an incubation in the light; dark green circles and closed squares incubation in darkness. Growth of *L. syltensis* DSM 22749^T^ in the complex medium SYMHC under air atmosphere (**A**) and in defined medium with 10 mM DL-malate as sole substrate under an initial headspace gas atmosphere of 20% (v/v) O_2_ (**B**). Growth of *C. halotolerans* DSM 23344^T^ in SYM medium supplemented with 0.5% (v/v) Tween 80 under air atmosphere (**C**) and in defined medium with 10 mM DL-malate as sole substrate under an initial headspace gas atmosphere of 20% (v/v) O_2_ (**D**). Growth of *P. rubra* DSM 19751^T^ in SYM medium under air atmosphere (**E**) and in defined medium with 10 mM DL-malate as sole substrate under an initial headspace gas atmosphere of 20% (v/v) O_2_ (**F**).

In order to determine to what extent the efficiency of light utilization varies between strains of the OM60/NOR5 clade we analysed the growth response under illumination and darkness in complex or defined media containing malate or pyruvate as principal carbon source. Upon incubation in complex media with malate and yeast extract as substrates the cell density in cultures of *L. syltensis* and *P. rubra* increased in light compared to growth in darkness (Figure 2A and E), whereas there was no measurable effect on biomass formation in *C. halotolerans* in SYM medium supplemented with 0.5% (w/v) Tween 80 (Figure 2C), although the overall level of produced photosynthetic pigments was similar in all three strains. Tween 80 was added to SYM medium, because it was found that it stimulated photosynthetic pigment production in cultures of *C. halotolerans*. The increase in growth yield (determined as dry weight) was 57% in *L. syltensis* and 21% in *P. rubra*. Mixotrophic growth of *P. rubra* was also tested in SYPHC medium containing pyruvate instead of malate in combination with yeast extract as substrate. However, in this medium no light-dependent increase of biomass formation was found (data not shown). Noteworthy, the growth yield of *P. rubra* in complex medium is much lower compared to *L. syltensis* and *C. halotolerans*, which could indicate that in *P. rubra* mainly pyruvate or malate were utilized for growth, but only a limited amount of the other carbon compounds that are present in yeast extract.

The growth response of the tested strains in defined media containing DL-malate as single substrate are shown in Figure 2B, D and F. In all three strains an increase in growth yield could be determined, which was on a dry weight basis around 14% in *L. syltensis*, 47% in *C. halotolerans* and 54% in *P. rubra*. Thus, in cultures of *L. syltensis* yeast extract stimulated not only the production of photosynthetic pigments, but also light-dependent mixotrophic growth. In *P. rubra* the stimulatory effect of light on growth with malate as sole carbon source could be partly due to an acceleration of the transportation of this substrate into the cell, which would explain that the generation time was shortened by half in cultures growing with malate in the light compared to darkness. Thus, in some strains of the OM60/NOR5 clade the energy generated from light could be partly used to facilitate the uptake of distinct substrates, instead of enhancing their assimilation as assumed for most aerobic anoxygenic photoheterotrophic bacteria studied so far [13].

For *L. syltensis* and *P. rubra* also growth curves with pyruvate were determined, because in both strains this substrate was more efficiently metabolized than malate (data not shown). However, no significant light-dependent increase in growth yield was found for *L. syltensis* and *P. rubra* upon incubation with pyruvate as sole carbon source, albeit photosynthetic pigments in amounts comparable to mixotrophically growing strains were produced, so that it can be assumed that during utilization of pyruvate no energy could be gained from the harvested light. Obviously, the metabolized substrate has a large impact on the efficiency of mixotrophic growth in members of the OM60/NOR5 clade, whereas the abundance of photosynthetic pigments does not correlate directly with the energy yield of photophosphorylation. Interestingly, no significant relationship between the cellular BChl *a* concentration and the photosynthetic competence in aerobic photoheterotrophic alphaproteobacteria could be found in a recent study by Sato-Takabe et al. [12] using a fluorescence induction and relaxation technique.

### Effect of light on pigment production is variable among strains

**Figure 3:**
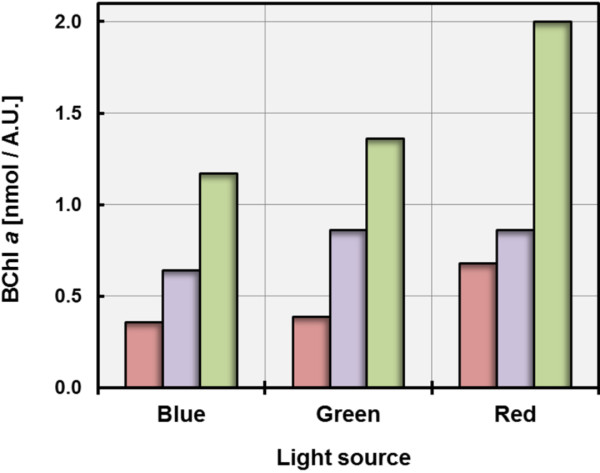
**Influence of the light source on the production of photosynthetic pigments.** The wavelength dependence of pigment production was tested under the following cultivation conditions, which allowed a high expression of the photosynthetic apparatus in the respective strains: *L. syltensis* DSM 22749^T^ was grown in SYMHC medium under an initial headspace gas atmosphere of 20% (v/v) O_2_, *C. halotolerans* DSM 23344^T^ in SYM medium containing 0.5% (v/v) Tween 80 under air atmosphere and *P. rubra* DSM 19751^T^ in defined medium containing 5 mM DL-malate under an initial headspace gas atmosphere of 12% (v/v) O_2_. The amount of produced BChl *a* is symbolized by red bars for *L. syltensis* DSM 22749^T^, blue bars for *C. halotolerans* DSM 23344^T^ and green bars for *P. rubra* DSM 19751^T^. Each experiment was performed in duplicate and the shown values represent means of two measurements.

As shown in Figure 2 the expression of photosynthetic pigments in *L. syltensis* and *P. rubra* was reduced by illumination with dim light (40 W tungsten incandescent bulb, ca. 1500 lux) compared to darkness. This represents a distinguishing trait to *C. halotolerans* and *C. litoralis*[15], but is similar to the effect described for several members of the *Roseobacter* clade in which synthesis of pigments is repressed even under conditions of low light intensities [21]. In *C. litoralis* sensitivity to light is restricted to blue light, which led to the assumption that a BLUF protein may participate in the regulation of the production of photosynthetic pigments [15]. In order to determine the effect of illumination with different wavelengths on the level of pigmentation of the strains used in this study, LED lamps emitting light of distinct wavelengths were used. It turned out that in contrast to *C. halotolerans* and *C. litoralis*, the synthesis of pigments in *L. syltensis* and *P. rubra* was not only repressed by illumination with blue light, but also by green LED light having a peak wavelength around 520 nm (Figure 3). This could explain the different effects of illumination by the 40 W tungsten incandescent light bulbs used in the growth experiments shown in Figure 2, which emit spectra with a maximum intensity at around 650 nm and contain only a negligible fraction of blue light (<470 nm). The different effects of light on the expression of photosynthetic pigments in aerobic gammaproteobacteria may have several reasons. Possible explanations could be some variation in the sensitivity of a light sensor interacting with the regulation of photosynthesis gene expression or a global repression of pigment synthesis due to oxidative stress caused by the interaction of blue-green light with the photosynthetic apparatus. In this regard it is interesting to note that in strains, which show a low sensitivity of pigment production to illumination the synthesis of unsaturated fatty acids seems to depend partly on the availability of oxygen [18]. Therefore, it is possible that in *C. litoralis* and *C. halotolerans* membrane bound fatty acid desaturases prevent the production of harmful singlet oxygen at the photosynthetic apparatus by using it immediately for the targeted introduction of double bonds in saturated fatty acids.

### The ratio of photosynthetic pigments depends on the redox conditions

**Figure 4:**
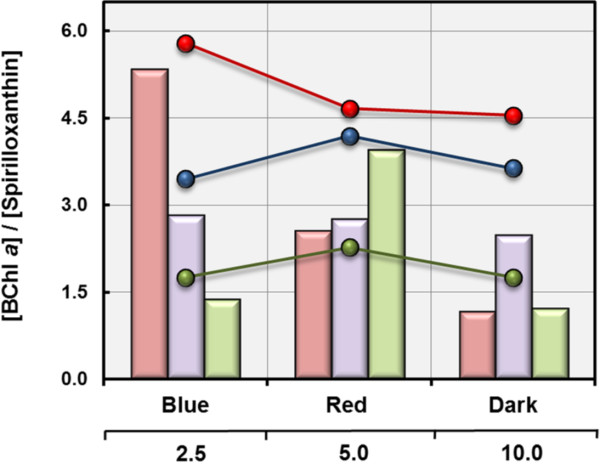
**Pigment stoichiometry in cells grown under various incubation conditions.** The determined photosynthetic pigment ratios are based on results obtained in the experiments shown in Figures 1B and 3. Bars illustrate pigment ratios obtained upon incubation with different amounts of a distinct carbon source and line graphs represent values determined upon growth under illumination with different light sources. Bars and graphs in red represent values of *L. syltensis* DSM 22749^T^, values of *C. halotolerans* DSM 23344^T^ are given in blue colour and values of *P. rubra* DSM 19751^T^ in green.

The pigment stoichiometry in *L. syltensis* varied widely and depended on the incubation conditions. Under conditions of a reducing environment (excess substrate, low oxygen concentrations, darkness) the determined BChl *a*/spirilloxanthin ratios were below one, whereas under oxidative stress (substrate limitation, high oxygen concentrations, illumination with blue light) the production of spirilloxanthin was inhibited and pigment ratios reached values above five (Figure 4). A similar interrelationship was previously found in *C. litoralis*[15], whereas the variation of pigment ratios in *C. halotolerans* and *P. rubra* did not correlate linearly with the environmental redox conditions. Especially, in *P. rubra* the BChl *a*/spirilloxanthin ratios reached higher values under optimal conditions for expression of the photosynthetic apparatus as under suboptimal conditions, irrespective of the environmental redox conditions being too high or too low for optimal pigment expression. It is noteworthy, that in these strains the observed variability of the pigment stoichiometry was independent of the total amount of produced photosynthetic pigments, which could indicate that the ratio and amount of produced photosynthetic pigments are controlled by two independent regulatory mechanisms.

### Possible influence of terminal oxidases on the regulation of pigment production

**Figure 5:**
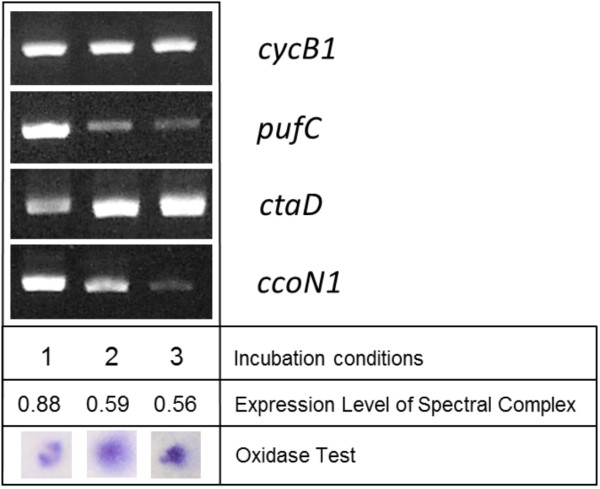
**Analyses of the transcription level of cytochromes and terminal oxidases in correlation with the expression of the photosynthetic apparatus in *C. litoralis *DSM 17192**^**T**^**.** Cultures were grown under the following incubation conditions: (1) with 6 mM malate as sole carbon source and an initial head space gas atmosphere of 6% (v/v) O_2_, (2) in SYPG complex medium at an initial head space gas atmosphere of 12% (v/v) O_2_, (3) with 3 mM sucrose at an initial head space gas atmosphere of 12% (v/v) O_2_. The expression level of the photosynthetic apparatus is given as A_880nm_/A_660nm_ values. The cytochrome *c* oxidase activity in whole cells was determined with N,N,N’,N’-tetramethyl-*p*-phenylenediamine (TMPD) as described previously [15]. The designation of analysed genes is explained in Table 1.

**Figure 6:**
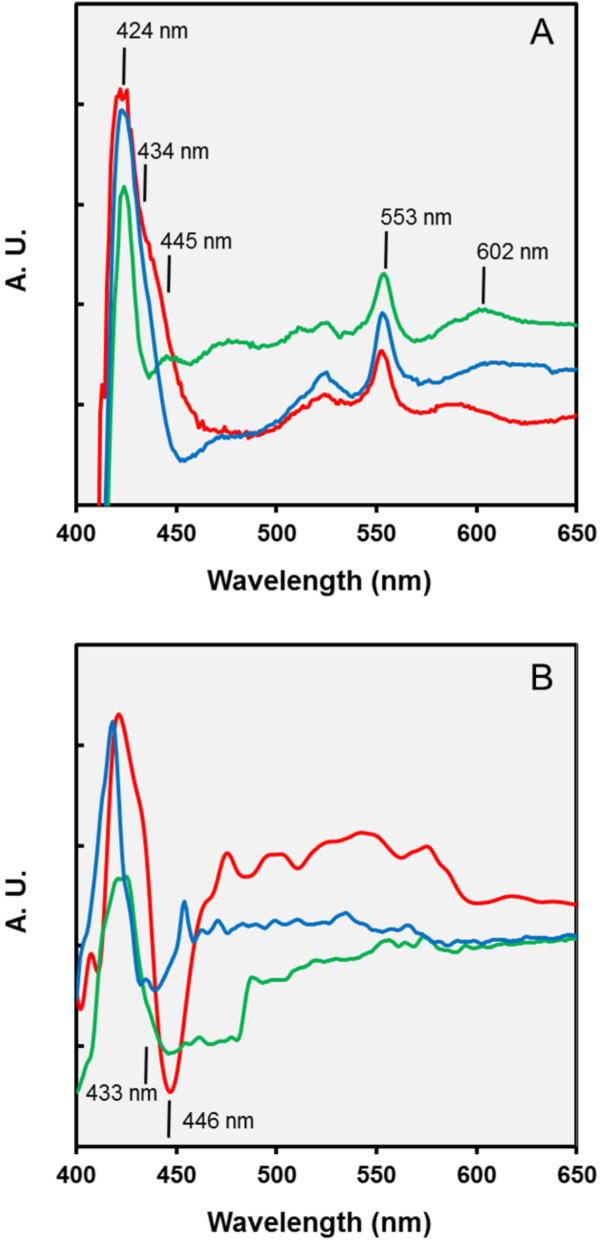
**Estimation of the expression of cytochromes in mixotrophically growing cells.** The expression of cytochromes was determined under the following cultivation conditions, which allowed a high expression of photosynthetic pigments and mixotrophic growth in the respective strains: *L. syltensis* DSM 22749^T^ was cultured in SYMHC medium under air atmosphere (red line), *C. halotolerans* DSM 23344^T^ (blue line) and *P. rubra* DSM 19751^T^ (green line) in defined medium containing 10 mM DL-malate at an initial head space gas atmosphere of 20% (v/v) O_2_. The position of distinct peaks of the spectra is indicated. A.U., arbitrary units of absorbance. **A**. Dithionite-reduced *minus* ferricyanide-oxidized redox difference spectra of extracts from whole cells solubilized with 0.3% (w/v) N,N-dimethyldodecylamine-N-oxide. Peaks at 424 and 553 nm indicate the presence of cytochrome *c* and the peak around 602 nm cytochrome *a*; shoulders in the Soret region at 434 and 445 nm the presence of cytochromes *b* and *a*, respectively. **B**. CO and dithionite-reduced *minus* dithionite-reduced difference spectra of intact cells. Troughs in the Soret region at 433 and 446 nm could indicate the binding of CO by heme *b* and *aa*_3_, respectively.

At least two different terminal oxidases, one belonging to the *cbb*_3_- and the other to the *caa*_3_-type, can be detected in all available genome sequences of photoheterotrophic members of the OM60/NOR5 clade [18], which therefore use a branched electron transport chain. In a previous study it was found that the activity of the *caa*_3_-type cytochrome *c* oxidase in *C. litoralis* appears to be repressed under conditions that stimulate the production of photosynthetic pigments [15], so that the *cbb*_3_-type oxidase becomes dominating. In subsequent experiments it turned out that part of the regulation takes place at the transcription level. By applying semiquantitative reverse transcriptase PCR less amounts of the mRNA encoding subunit I of the *caa*_3_ oxidase (*ctaD* gene) was detected in strongly pigmented cells compared to non-pigmented cells (Figure 5). Provided that the differential expression of terminal oxidases plays a role in the regulation of the photosynthetic pigments production in members of the OM60/NOR5 clade, a similar effect should be also detectable in cells of *L. syltensis*, *C. halotolerans* and *P. rubra*. However, albeit some variation of the total quantity of cytochromes depending on the incubation conditions was found, no correlation of the abundance of the photosynthetic apparatus with the prevalence of a distinct oxidase could be demonstrated in the analysed strains, at least by the evaluation of data obtained by redox difference spectroscopy (Figure 6). Only in cells of *C. halotolerans* cytochromes containing heme *b* could be clearly detected besides the dominating c-type cytochromes by a shoulder around 434 nm in dithionite-reduced minus ferricyanide-oxidized redox difference spectra (Figure 6A) and a *cb*-type oxidase became apparent in CO and dithionite-reduced *minus* dithionite reduced difference spectra (Figure 6B). On the other hand, in fully pigmented cells of *L. syltensis* and *P. rubra* a *caa*_3_-type oxidase seems to be prevalent, which is indicated by a trough around 446 nm in CO and dithionite-reduced *minus* dithionite-reduced difference spectra (Figure 6B). However, this does not exclude the possibility that a *cbb*_3_-type oxidase is expressed constitutively in small amounts in these strains and participates in regulatory pathways by sensing the electron flow to oxygen.

### Complex substrates, the stringent response and the concept of oligotrophy

In *L. syltensis* pigment expression and photophosphorylation could be stimulated by the addition of yeast extract, whereas in *P. rubra* and *C. litoralis* complex nutrients had a negative effect. An ambiguous situation was obtained in *C. halotolerans*, because pigment expression could be stimulated by the combination of yeast extract and Tween 80, whereas yeast extract alone had a negative effect. It is known that yeast extract contains various compounds of different reduction levels, hence it is possible that *L. syltensis* utilizes other yeast extract derived carbon sources than *C. litoralis* or that different metabolic pathways are used for the same substrates leading to different intracellular redox states affecting regulatory pathways controlling pigment production. An excess of complex nutrients influences not only the level of pigmentation, but affects also the tendency for aggregation and cell morphology of the studied strains [18] and it seems that the intensity of these effects correlates with the observed repression of pigment production, which is most pronounced in *C. litoralis*[15] and *P. rubra*. Thus, this finding implies the participation of a global regulatory network in the expression of photosynthesis genes in some members of the OM60/NOR5 clade. In most gammaproteobacteria a deprivation of amino acids or carbon starvation leads to a global change in gene expression known as stringent response, which is mediated by the enzymes RelA and SpoT [22]. In fact, a stimulating effect of the guanosine 3′, 5′-bisdiphosphate (ppGpp) related stringent response on phototrophic growth of the alphaproteobacterium *Rhodobacter capsulatus* has been revealed [23]. Hence, various effects of the stringent response on the expression of photosynthetic pigments in members of the OM60/NOR5 clade could offer an explanation for the observed differences upon growth in nutrient rich complex media.

The observation that supplementation of media with complex nutrients in amounts of around 1 g/l stimulated the production of photosynthetic pigments in several strains of the OM60/NOR5 clade contradicts their designation as obligate oligotrophic photoheterotrophs as originally proposed by Cho et al. [16]. In general, a distinction of marine bacteria in obligate or facultative oligotrophs on the one hand and copiotrophs on the other hand is quite difficult to verify. According to the definition of Ishida et al. [24] obligate oligotrophs cannot grow in media containing above around 0.3 g/l carbon, which would be an inherent characteristic of these strains. However, inhibition of growth on nutrient rich media may have several reasons, especially if strains are analysed that were freshly isolated from the environment. In most cases the optimal growth conditions and traits of novel isolates are unknown, so that a lack of growth in nutrient rich media may be caused by impurities of highly concentrated substrates, harmful metabolic endproducts, activation of lysogenic phages or simply inappropriate incubation conditions. It can be assumed that most bacteria isolated from seawater inhabit oligotrophic niches, so that the observed differences of various marine bacteria in the response to high nutrient concentrations could be just based on variations of the time period required to adapt to the elevated nutrient concentrations used in laboratory media to achieve high growth yields. The existing distinguishable growth response of most members of the *Roseobacter* clade on the one hand, which are easily isolated and cultivated on nutrient rich media and the more fastidious representatives of the OM60/NOR5 clade on the other hand could thus be based on effects reflecting different strategies of gene regulation and adaptation. A similar conclusion was drawn earlier by Schut et al. [25], who stated that obligate oligotrophy can be understood as a transient characteristic observed in cells that are taken directly from an extremely substrate-limited natural environment.

## Conclusions

We propose that the specific regulation of photosynthesis genes in members of the OM60/NOR5 clade depends on a redox-sensitive repressor encoded by the *ppsR* gene, which has been detected within the photosynthesis superoperon in most genome-sequenced photoheterotrophic proteobacteria [18,26,27], including *C. litoralis*, *L. syltensis* and *P. rubra* (unpublished data). The PpsR dependent regulation could be either independent from other involved regulatory pathways that influence pigment expression or PpsR represents a terminal effector that interacts with various sensors for diverse environmental stimuli, like for instance a single domain BLUF protein sensing blue light or a yet unknown sensor of membrane-bound lipoquinone reduction. Recently, it could be shown that the PpsR repressor of *Rhodobacter capsulatus* has heme sensing activity [28]. According to this study the binding of free heme to PpsR has an influence on operator affinity, which depends on the target sequence. This effect could explain the linear dependence of the BChl *a*/spirilloxanthin ratio on the cellular redox state in cells of *L. syltensis* and *C. litoralis*. A discrimination between operators controlling bacteriochlorophyll and carotenoid synthesis would be possible, if in *L. syltensis* and *C. litoralis* the proportion of PpsR with bound heme is influenced by the cellular redox state.

In addition to the postulated specific regulation by a redox-sensitive regulatory protein a signalling pathway controlling global gene expression might be involved in the expression of photosynthesis genes. An indication for two different modes of regulation could be that in *L. syltensis* and *C. litoralis* the ratio of BChl *a* to spirilloxanthin correlates reliably with the estimated cellular redox state, but is quite independent of the overall level of pigment expression (Figure 4). The proposed global regulation of pigment production could be based for example on the activity of a *cbb*_3_-type oxidase which has been shown to control the production of photosynthetic pigments in a *Rhodobacter* species [29]. Alternatively, the second messenger (p)ppGpp responsible for inducing and maintaining the stringent response in most gammaproteobacteria could promote the expression of photosynthesis genes in response to the limited availability of complex nutrients.

Furthermore, our results indicate that the mechanisms regulating pigmentation in strains from different lineages of aerobic photoheterotrophic gammaproteobacteria are quite similar to the well-studied regulatory pathways in facultatively anaerobic photoheterotrophic purple bacteria [30]. In both cases the intracellular redox state plays a major role in pigment expression and photoheterotrophic growth [19,20]. The only main difference to the regulation in facultative anaerobic photosynthetic purple bacteria appears to be the absence of an energy-intensive redox-balancing system based on the fixation of carbon dioxide or nitrogen (so far no genes encoding enzymes of both pathways were detected in obligately aerobic anoxygenic photoheterotrophic bacteria), which prevents the decrease of the intracellular redox state to suboptimal levels for photosynthesis under reducing conditions. In conclusion, we postulate that in obligately aerobic anoxygenic photoheterotrophic gammaproteobacteria a decrease of the intracellular redox state is used to sense a surplus of suitable carbon sources, which makes a photosynthetic apparatus redundant. On the other hand, the type of regulation in most BChl *a*-containing members of the *Roseobacter* clade seems to be fundamentally different, because in these species the expression level of the photosynthetic apparatus is almost exclusively controlled by light.

## Methods

### Used strains, media and cultivation conditions

The following reference strains were taken from the DSMZ culture collection and used in this study: *Luminiphilus syltensis* DSM 22749^T^, *Chromatocurvus halotolerans* DSM 23344^T^ (= EG19^T^), *Pseudohaliea rubra* DSM 19751^T^ (= CM41_15a^T^) and *Congregibacter litoralis* DSM 17192^T^ (= KT71^T^). *Pseudohaliea rubra* CM41_15a^T^ was deposited in the DSMZ by the Laboratoire Arago, Université Pierre et Marie Curie (Banyuls-sur-Mer, France) under the conditions of a Material Transfer Agreement. For routine cultivation all strains were grown in SYPHC medium at 28°C [15]. Replacing of pyruvate in SYPHC medium with 10 mM DL-malate resulted in SYMHC medium. SYM medium was obtained, if the supplementary amino acids L-histidine and L-cysteine were omitted. The preparation of defined media for growth on single carbon sources and the generation of various gas atmospheres in batch cultures has been described elsewhere [15,18]. A 40 W incandescent bulb was used as light source for the determination of growth curves in the light. For the illumination of cultures with light of distinct wavelengths LED lamps were used emitting blue, green and red visible light with peak wavelengths of 627, 518 and 466 nm, respectively. All used chemicals were obtained from Sigma-Aldrich (Taufkirchen, Germany) and complex nutrients from DIFCO BBL (Becton Dickinson; Heidelberg, Germany).

### Determination of growth, cellular pigmentation and cytochromes

The absorbance values of growing cultures were determined in a Thermo Scientific BioMate 6 split beam UV/visible spectrophotometer using 1 cm light path disposable cuvettes and water as blank. The A_660nm_ reading was used to estimate the cell density. The cellular dry weight of grown cultures was determined by overnight freeze-drying of cell pellets harvested by centrifugation. Expression of the light-harvesting complex in *L. syltensis* was estimated by determining the A_870nm_ to A_660nm_ ratio, for cultures of *C. litoralis* and *C. halotolerans* a ratio of A_880nm_ to A_660nm_ was used and for *P. rubra* a ratio of A_820nm_ to A_660nm_. Photosynthetic pigments were extracted from wet cell pellets using a mixture of acetone/methanol (7:2) as described previously [15]. The concentrations of bacteriopheophytin *a*, bacteriochlorophyll *a* and spirilloxanthin in the acetone/methanol extracts were determined from the absorbance values obtained at 747, 771 and 475 nm, respectively, using the spectral reconstruction method of van der Rest and Gingras [31].

The detection and identification of various cytochrome types was done as reported previously [15].

### Semiquantitative detection of transcripts using PCR

RNA was isolated from cultures of *C. litoralis* DSM 17192^T^ that were grown to early stationary phase under various incubation conditions. A culture volume equivalent to a cell suspension of one ml with an A_660nm_ of approx. 1.0 was diluted with two volumes of RNAprotect Bacteria Reagent (Qiagen; Hilden, Germany), then cells were harvested by centrifugation. The cell pellet was frozen at −20°C overnight and subsequently used for the extraction of RNA using the Qiagen RNeasy Midi Kit including the optional on-column DNase digestion. In most cases this procedure yielded ca. 10 μg of extracted total RNA as determined by photometric analysis at 260 nm. Despite the applied on-column DNase treatment small quantities of genomic DNA could still be detected in the purified RNA samples by PCR amplification. Hence, an additional DNase treatment in solution was applied to obtain DNA-free RNA.

Reverse transcriptase-PCR (RT-PCR) of mRNA was performed with the OneStep RT-PCR kit of Qiagen following the instructions given by the manufacturer and using 0.5 μg of total RNA. Gene-specific primers are listed in Table 1 and the following thermal cycler conditions were used for amplification: reverse transcription at 50°C for 30 min, an initial step at 95°C for 15 min and then 30 cycles at 94°C for 30 s, 58°C for 1 min and 72°C for 1 min. At the end a postelongation at 72°C for 5 min was carried out. RT-PCR products were visualized using the FlashGel electrophoresis system with DNA Cassettes (2.2% agarose) from Lonza (Verviers, Belgium) and a Kodak EDAS 290 imaging system. Normalization of mRNA levels was performed using specific *rpoZ* primers (Table 1), which amplify the omega subunit of the RNA polymerase, a housekeeping gene that seems to be expressed constitutively in a *Rhodobacter* species [32].

**Table 1:** **Oligonucleotides used for the amplification of gene fragments from *C. litoralis *DSM 17192**^**T **^**with PCR or semiquantitative RT-PCR**

**Primer**	**Sequence (5′-3′)**	**T**_**a **_**(°C)**	**Protein encoded by the target gene**	**Product size (bp)**
**KT71 rpoZ-F**	CAT CAC TTC GGC GAG TTC TT	58	RNA polymerase omega subunit	223
**KT71 rpoZ-R**	AGA AGA TTG CCT TGA GTC CG			
**KT71 cycB1-F**	GAC AGT CGG TTT GAT TGC AG	58	Cytochrome *c*_5_	204
**KT71 cycB1-R**	CAT GCG GTG TTG TAA GTT GC			
**KT71 pufC-F**	AAG CAG ACC GAG TGG ACC TA	58	Photosynthetic reaction centre cytochrome *c* subunit	373
**KT71 pufC-R**	GTG CCT TCT CAG ACT CCG TC			
**KT71 ctaD-F**	ATA TCC ACT TTG GCA CCA GC	58	*Caa*_3_-type cytochrome *c* oxidase subunit 1	409
**KT71 ctaD-R**	GTG AAG AGC ACA AGG AAG CC			
**KT71 ccoN1-F**	CTT ATC ACC GTC GTC TGG GT	58	*Cbb*_3_-type cytochrome oxidase CcoN subunit	392
**KT71 ccoN-R**	GTG TAG TGC AGG TGG TGT GG			

T_a_ indicates the annealing temperature used in the PCR reaction.

## Competing interests

The authors declare that they have no competing interests.

## Authors’ contributions

SS developed the study concept. SS conceived and designed a majority of the experiments. SS and TR performed the experiments. SS wrote the paper. Both authors read and approved the final manuscript.

## References

[B1] Jiao N Zhang Y Zeng Y Hong N Liu R Chen F Wang P Distinct distribution pattern of abundance and diversity of aerobic anoxygenic phototrophic bacteria in the global ocean Environ Microbiol 2007 9 3091 3099 10.1111/j.1462-2920.2007.01419.x 17991036

[B2] Lami R Cottrell MT Ras J Ulloa O Obernosterer I Claustre H Kirchman DL Lebaron P High abundances of aerobic anoxygenic photosynthetic bacteria in the South Pacific Ocean Applied Environ Microbiol 2007 73 4198 4205 10.1128/AEM.02652-06 PMC1932784 17496136

[B3] Yutin N Suzuki MT Teeling H Weber M Venter JC Rusch DB Béjà O Assessing diversity and biogeography of aerobic anoxygenic phototrophic bacteria in surface waters of the Atlantic and pacific oceans using the global ocean sampling expedition metagenomes Environ Microbiol 2007 9 1464 1475 10.1111/j.1462-2920.2007.01265.x 17504484

[B4] Brinkhoff T Giebel H-A Simon M Diversity, ecology, and genomics of the *Roseobacter* clade: a short overview Arch Microbiol 2008 189 531 539 10.1007/s00203-008-0353-y 18253713

[B5] Yan S Fuchs BM Lenk S Harder J Wulf J Jiao NZ Amann R Biogeography and phylogeny of the NOR5/OM60 clade of *Gammaproteobacteria* Syst Appl Microbiol 2009 32 124 139 10.1016/j.syapm.2008.12.001 19216045

[B6] Jiao N Zhang F Hong N Significant roles of bacteriochlorophyll *a* supplemental to chlorophyll *a* in the ocean ISME J 2010 4 595 597 10.1038/ismej.2009.135 20010633

[B7] Kolber ZS Plumley FG Lang AS Beatty JT Blankenship RE VanDover CL Vetriani C Koblížek M Rathenberg C Falkowski PG Contribution of aerobic photoheterotrophic bacteria to the carbon cycle in the Ocean Science 2001 292 2492 2495 10.1126/science.1059707 11431568

[B8] Iba K Takamiya K Action spectra for inhibition by light of accumulation of bacteriochlorophyll and carotenoid during aerobic growth of photosynthetic bacteria Plant Cell Physiol 1989 30 471 477

[B9] Yurkov VV van Gemerden H Impact of light/dark regimen on growth rate, biomass formation and bacteriochlorophyll synthesis in *Erythromicrobium hydrolyticum* Arch Microbiol 1993 159 84 89 10.1007/BF00244268

[B10] Biebl H Wagner-Döbler I Growth and bacteriochlorophyll *a* formation in taxonomically diverse aerobic anoxygenic phototrophic bacteria in chemostat culture: influence of light regimen and starvation Proc Biochem 2006 41 2153 2159 10.1016/j.procbio.2006.06.029

[B11] Koblížek M Mlcousková J Kolber Z Kopecký J On the photosynthetic properties of marine bacterium COL2P belonging to *Roseobacter* clade Arch Microbiol 2010 192 41 49 10.1007/s00203-009-0529-0 19949940

[B12] Sato-Takabe Y Hamasaki K Suzuki K Photosynthetic characteristics of marine aerobic anoxygenic phototrophic bacteria *Roseobacter* and *Erythrobacter* strains Arch Microbiol 2012 194 331 341 10.1007/s00203-011-0761-2 22033765

[B13] Hauruseu D Koblížek M Influence of light on carbon utilization in aerobic anoxygenic phototrophs Applied Environ Microbiol 2012 78 7414 7419 10.1128/AEM.01747-12 PMC3457121 22885759

[B14] Tomasch J Gohl R Bunk B Diez MS Wagner-Döbler I Transcriptional response of the photoheterotrophic marine bacterium *Dinoroseobacter shibae* to changing light regimes ISME J 2011 5 1957 1968 10.1038/ismej.2011.68 21654848 PMC3223308

[B15] Spring S Lünsdorf H Fuchs BM Tindall BJ The photosynthetic apparatus and its regulation in the aerobic gammaproteobacterium *Congregibacter litoralis* gen. nov., sp. nov PLoS One 2009 4 3 e4866 10.1371/journal.pone.0004866 19287491 PMC2654016

[B16] Cho J-C Stapels MD Morris RM Vergin KL Schwalbach MS Givan SA Barofsky DF Giovannoni SJ Polyphyletic photosynthetic reaction centre genes in oligotrophic marine *Gammaproteobacteria* Environ Microbiol 2007 9 1456 1463 10.1111/j.1462-2920.2007.01264.x 17504483

[B17] Csotonyi JT Stackebrandt E Swiderski J Schumann P Yurkov V *Chromocurvus halotolerans* gen. nov., sp. nov., a gammaproteobacterial obligately aerobic anoxygenic phototroph, isolated from a Canadian hypersaline spring Arch Microbiol 2011 193 573 582 10.1007/s00203-011-0698-5 21479531

[B18] Spring S Riedel T Spröer C Yan S Harder J Fuchs BM Taxonomy and evolution of bacteriochlorophyll *a*-containing members of the OM60/NOR5 clade of marine gammaproteobacteria: Description of *Luminiphilus syltensis* gen. nov., sp. nov., reclassification of *Haliea rubra* as *Pseudohaliea rubra gen. nov.,* comb. nov., and emendation of *Chromatocurvus halotolerans* BMC Microbiol 2013 13 118 10.1186/1471-2180-13-118 23705883 PMC3679898

[B19] Grammel H Ghosh R Redox-state dynamics of ubiquinone-10 imply cooperative regulation of photosynthetic membrane expression in *Rhodospirillum rubrum* J Bacteriol 2008 190 4912 4921 10.1128/JB.00423-08 18487324 PMC2446998

[B20] Laguna R Tabita FR Alber BE Acetate-dependent photoheterotrophic growth and the differential requirement for the Calvin-Benson-Bassham reductive pentose phosphate cycle in *Rhodobacter sphaeroides* and *Rhodopseudomonas palustris* Arch Microbiol 2011 193 151 154 10.1007/s00203-010-0652-y 21104179

[B21] Yurkov VV Beatty JT Aerobic anoxygenic phototrophic bacteria Microbiol Mol Biol Rev 1998 62 695 724 9729607 10.1128/mmbr.62.3.695-724.1998 PMC98932

[B22] Braeken K Moris M Daniels R Vanderleyden J Michiels J New horizons for (p)ppGpp in bacterial and plant physiology Trends Microbiol 2006 14 45 54 10.1016/j.tim.2005.11.006 16343907

[B23] Masuda S Bauer CE Null mutation of HvrA compensates for loss of an essential relA/spoT-like gene in *Rhodobacter capsulatus* J Bacteriol 2004 186 235 239 10.1128/JB.186.1.235-239.2004 14679243 PMC303453

[B24] Ishida Y Eguchi M Kadota H Existence of obligately oligotrophic bacteria as a dominant population in the south China Sea and the west Pacific Ocean Mar Ecol Prog Ser 1986 30 197 203

[B25] Schut F Prins R Gottschal J Oligotrophy and pelagic marine bacteria: facts and fiction Aquat Microb Ecol 1997 12 177 202

[B26] Elsen S Jaubert M Pignol D Giraud E PpsR: a multifaceted regulator of photosynthesis gene expression in purple bacteria Mol Microbiol 2005 57 17 26 10.1111/j.1365-2958.2005.04655.x 15948946

[B27] Zheng Q Zhang R Koblížek M Boldareva EN Yurkov V Yan S Jiao N Diverse arrangement of photosynthetic gene clusters in aerobic anoxygenic phototrophic bacteria PLoS One 2011 6 e25050 10.1371/journal.pone.0025050 21949847 PMC3176799

[B28] Yin L Dragnea V Bauer CE PpsR, a regulator of heme and bacteriochlorophyll biosynthesis, is a heme-sensing protein J Biol Chem 2012 287 13850 13858 10.1074/jbc.M112.346494 22378778 PMC3340169

[B29] Oh J-I Kaplan S Redox signaling: globalization of gene expression EMBO J 2000 19 4237 4247 10.1093/emboj/19.16.4237 10944106 PMC302043

[B30] Bauer CE Elsen S Swem LR Swem DL Masuda S Redox and light regulation of gene expression in photosynthetic prokaryotes Phil Trans R Soc Lond B Biol Sci 2003 358 147 154 10.1098/rstb.2002.1189 12594923 PMC1693112

[B31] Van der Rest M Gingras G The pigment complement of the photosynthetic reaction center isolated from *Rhodospirillum rubrum* J Biol Chem 1974 249 6446 6453 4214257

[B32] Pappas CT Sram J Moskvin OV Ivanov PS Mackenzie RC Choudhary M Land ML Larimer FW Kaplan S Gomelsky M Construction and validation of the *Rhodobacter sphaeroides* 2.4.1 DNA microarray: transcriptome flexibility at diverse growth modes J Bacteriol 2004 186 4748 4758 10.1128/JB.186.14.4748-4758.2004 15231807 PMC438620

